# Multi-Pixel Photon Counters for Optofluidic Characterization of Particles and Microalgae

**DOI:** 10.3390/bios5020308

**Published:** 2015-06-12

**Authors:** Pouya Asrar, Marta Sucur, Nastaran Hashemi

**Affiliations:** Department of Mechanical Engineering, Iowa State University, Ames, IA 50011, USA; E-Mails: pouya@gatech.edu (P.A.); msucur@iastate.edu (M.S.)

**Keywords:** optofluidics, microfluidics, multi-pixel photon counter

## Abstract

We have developed an optofluidic biosensor to study microscale particles and different species of microalgae. The system is comprised of a microchannel with a set of chevron-shaped grooves. The chevrons allows for hydrodynamic focusing of the core stream in the center using a sheath fluid. The device is equipped with a new generation of highly sensitive photodetectors, multi-pixel photon counter (MPPC), with high gain values and an extremely small footprint. Two different sizes of high intensity fluorescent microspheres and three different species of algae (*Chlamydomonas reinhardtii* strain 21 gr, *Chlamydomonas suppressor*, and *Chlorella sorokiniana*) were studied. The forward scattering emissions generated by samples passing through the interrogation region were carried through a multimode fiber, located in 135 degree with respect to the excitation fiber, and detected by a MPPC. The signal outputs obtained from each sample were collected using a data acquisition system and utilized for further statistical analysis. Larger particles or cells demonstrated larger peak height and width, and consequently larger peak area. The average signal output (integral of the peak) for *Chlamydomonas reinhardtii* strain 21 gr, *Chlamydomonas suppressor*, and *Chlorella sorokiniana* falls between the values found for the 3.2 and 10.2 μm beads. Different types of algae were also successfully characterized.

## 1. Introduction

Microfluidic technology is being developed with the purpose of quantifying properties of system particles in environmental studies or clinical diagnostics [1,2,3,4,5]. Flow cytometry has attracted considerable research attention in recent years due to its high-throughput capability in performing both quantitative and qualitative analyses of cells or particles. Employing hydrodynamic focusing, a single-file stream of cells is created. Light scatter and fluorescence optical signals are collected once the focused cell or particle passes through a laser beam. A single-layer flow cytometer capable of multi-parametric particle analysis has been reported [6]. The design facilitates a three-dimensional hydrodynamic focusing by ‘microfluidic drifting’ and on-chip detection simultaneously. Surface patterns such as chevron grooves are also used to hydrodynamically focus the core stream and interrogate each sample at four different wavelengths [7,8,9].

The size of flow cytometers has decreased significantly over time even when imaging capabilities such as digital microscopy on a cellphone [10] were incorporated to the systems. However, the complexity of their large optical trains, sensitivity, and power requirement has prevented them from being effectively used in harsh environments or portable point-of-care diagnostics [11,12].

In this study, we report the design and development of an optofluidic cytometer equipped with multi-pixel photon counters (MPPCs)—a new generation of photodetectors—as part of the system optical train. Avalanche photo diodes (APDs) and photomultiplier tubes (PMTs) are popular types of photo detectors. However, PMTs have the disadvantage of being too large and APDs have very small gains [13]. With research in flow cytometry moving towards developing devices with small footprints, a large photodetector such as a PMT could not be an appropriate choice for a portable system.

A handheld prototype of a flow cytometer which uses a PIN photodiode rather than a PMT is reported by Kiesel *et al.* [12]. However the detection limit was about 200 molecules of equivalent phycoerythrin for 2 μm Rainbow calibration beads. It was reported that using avalanche photodiode could improve the sensitivity of the system. Kotz *et al.*, designed a portable and lightweight device that utilizes inertial focusing and waveguides embossed into an optical-grade thermoplastic to direct light for excitation [11]. However, employing photomultiplier tubes to collect side scatter prevents significant size reduction in the design. Moreover, a comparison study between the photon detection efficiency of the PMTs and MPPCs by Yokoyama *et al.*, revealed that the efficiency of MPPCs for green light is at least twice of the efficiency of the PMTs [14]. The quantum efficiency of MPPC was reported to be twice of the PMT quantum efficiency [15]. Vacheret *et al.*, have performed a comprehensive study on measuring the dark noise level of MPPCs as well as their photon detection efficiency. They have implemented a simulation to predict the energy resolution of the MPPCs and reported the dark rate distribution *versus* temperature for different values of overvoltage. For sensitive area of 1.3 × 1.3 mm^2^, the photodetection efficiency was reported to be 28% when the photodetector was illuminated with a 515 nm light. It was also reported that the probability of hitting multiple photons to the same pixel at the same time is small, confirming the high time resolution of MPPCs [16]. In another study by Soto *et al.*, the main features of MPPC array channels were investigated at several different temperatures. In this study, gain, breakdown voltage, photon detection efficiency, optical crosstalk and dark rate for each of the MPPC array channels at each temperature were obtained [17].

MPPCs have very small footprint and can be integrated into the already developed compact microfluidic system [8] so that the whole analytical device meets the goal of being portable. Using MPPCs as the photodetectors in our design is the major difference between our system and the previous generations of the flow cytometers. MPPCs also have higher gain compared to the APDs. This would reduce the signal to noise ratio. In other words having higher gain in photodetection unit of the system facilitates the production of a more distinct signal output for each cell or particle. In addition to high sensitivity and low noise, MPPCs are not sensitive to magnetic fields and are able to detect photon packages in very small numbers [18]. Using the MPPC photodetectors in our optofluidic device, we were able to develop a system that is more compact and has higher resolution compared to earlier generations of flow cytometers. We have validated the performance of the device through discrimination and enumeration of beads and microalgae.

## 2. Materials and Methods

### 2.1. Microfluidics

The mold of the microfluidic channel was fabricated using standard photolithography techniques. The chip consists of two channels for sheath flow and one middle channel for core flow. The microchannel is made of polydimethylsiloxane (PDMS). Both sheath and sample streams are introduced into the channel using a bidirectional syringe pump (Cole-Parmer, Vernon Hills, IL, USA) at 200 and 10 μL/min, respectively. The height and width of the channel are 130 and 390 μm. Arrays of chevrons are designed and fabricated on both top and bottom of the microchannel at 100 μm width and 65 μm height. The design includes a set of reversed chevrons downstream of the interrogation region for unsheathing.

The core solution contains microbeads/cells with concentration of 50 beads per microliter. The beads are a fluorescent sky blue color (Spherotech Inc., Lake Forest, IL, USA), with two different diameters of 3.2 and 10.2 μm, and an excitation peak of 635 nm. In this study, *Chlamydomonas reinhardtii* strain 21 gr, *Chlamydomonas suppressor*, and *Chlorella sorokiniana* algae were introduced into the microchannel to investigate their size and granularity. In the experiments, filtered DI water is used as sheath fluid to avoid adding impurities.

### 2.2. Optics and Electronics

**Figure 1:**
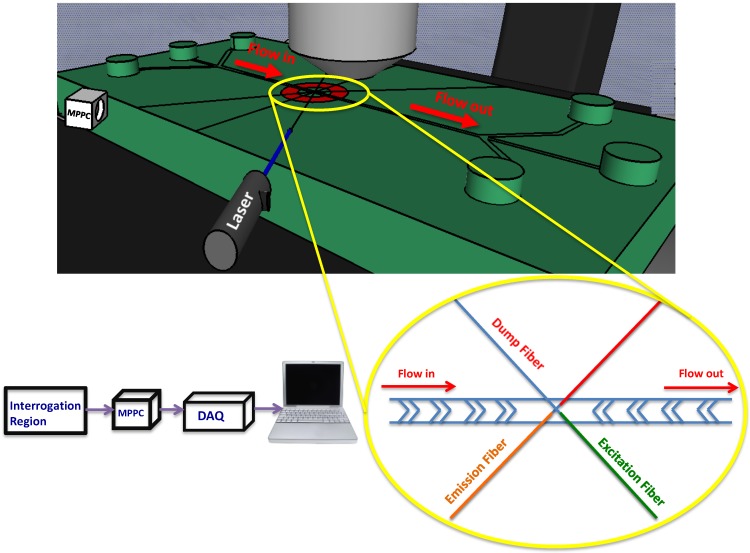
A schematic of the microfluidic chip with integrated optics. The laser beam is transferred through a fiber to the interrogation region where the microparticles pass through. An multi-pixel photon counter (MPPC) collects scatted light from the microparticles transferred by the emission fiber. A data acquisition unit collects the analog data from the photodetector and sends it to a desktop computer for further analysis.

Multimode optical fibers (fiber instrument sales Inc., Oriskany, NY, USA) were utilized as excitation and emission carriers. Fibers were precisely inserted into the channel to excite the samples in a definite wavelength and collect scattered light (Figure 1). The excitation light was provided using a 635 nm red laser. The emission fibers are perpendicular to the excitation fiber and are responsible for carrying the light from microbeads to the photodetection unit of the system. The emission fibers are perfectly mounted in the same plane as the excitation fiber to receive the highest intensity of light emitted from the samples. The MPPC is located in front of emission fibers so it is able collect the emitted light. We used a ceramic MPPC with an effective photosensitive area of 3 × 3 mm^2^ to detect the forward light scattered from microparticles.

A data acquisition (DAQ) unit (NI USB-6351, National Instrument, Austin, TX, USA) was employed in the system to collect the analog data from the photodetection unit and send it to a desktop computer. An analog input port of the DAQ device was specified for collecting data points received from particles/cells. LabVIEW software was coupled with the DAQ unit to monitor and visualize the sampling process on the computer. For each set of data, an independent channel was designed in the LabVIEW block diagram in order to record the results for each bead or cell separately. The sampling rate frequency was set to 100,000 samples per second on LabVIEW to make sure that enough data points are obtained from samples for accurate analysis. First, the current output from the MPPC was very low and out of detection range for DAQ device. Therefore, a circuit was designed to amplify and enhance the original signal output obtained from MPPC. Electrical components such as op-amps were installed in the system to increase the obtained voltage from samples to an acceptable range for the data acquisition unit. Also, a capacitor was utilized in the electrical circuit to run the voltage output with delay so the signal could be matched with the normal signal input of the DAQ device. The MPPC was supplied with 70 V by a power supply (HY3005-3 DC Power Supply, San Jose, CA, USA). The background signal (noise) for the MPPC could be adjusted by changing the supply voltage. A bypass filter (700 ± 10 nm, Thorlabs Inc., Newton, NJ, USA) was located between the emission fiber and the MPPC module. The filter canceled noise signals to record signal output only related to the sample response. A threshold value was set to remove most of the noise signal outputs.

## 3. Results and Discussion

### 3.1. COMSOL Modeling

COMSOL Multiphysics software was used to simulate the microchannel and confirm the experimental results. Only one fourth of the channel was simulated and analyzed by COMSOL to reduce the calculation time [19]. The velocity distribution was simulated by solving Navier-Stokes at a steady state condition. The concentration distribution along the channel was determined using the diffusive transport section. The sheath and sample flow rates were set to 200 and 10 μL/min. These values are identical to our experiments and allow for comparisons. The diffusivity was fixed at 10^9^ m^2^/s [1]. A structured meshing pattern offers a homogeneous pattern on all surfaces and results in more accurate analysis. The horizontal and vertical surfaces have triangular and rectangular features of meshing. Due to symmetry, the velocity and concentration distribution along the channel was only simulated for half of the microchannel to decrease the computational time. The results of the laminar flow simulation (velocity distribution) were used as an initial condition for the transport of diluted species (concentration distribution).

**Figure 2:**
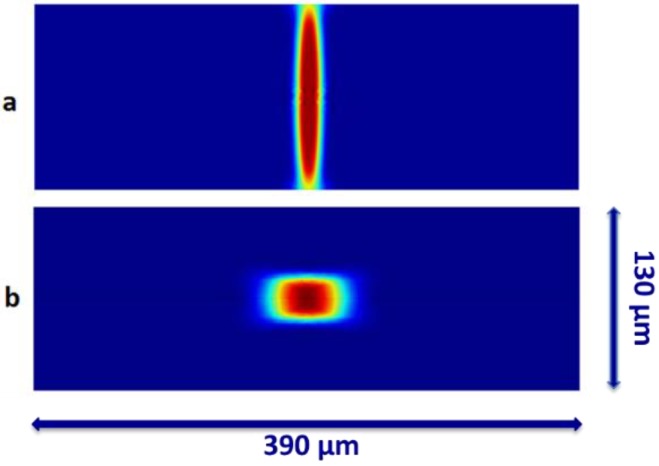
The concentration distribution along the channel for the cross section at: (**a**) before the arrays of chevrons; (**b**) after the forth chevron groove. The sheath stream focuses the core stream vertically before the chevrons. As the flow passes through the chevrons, the sheath stream focus the core stream horizontally in the middle of the channel. The concentration of sheath and sample streams were assumed to be 0 mol/m^3^ (blue) and 1 mol/m^3^ (red).

Figure 2 shows the concentration distribution along the channel. The concentration of sheath and sample streams were assumed to be 0 mol/m^3^ (blue) and 1 mol/m^3^ (red). The region in the center of the microchannel (indicated by red) shows the confined region where the beads could locate after passing through the chevrons. As the beads are present only in the sample stream, the red region in Figure 2 shows how the sheath stream focuses the sample in the center of the microchannel for optical sensing. The beads can be positioned in any point in the focused region at the time of optical sensing. The simulations are employed to optimize the design of the microchannel for the beads to receive the maximum excitation from the laser light at the focused region. The velocity profile was assumed to be fully developed at the entrance of the channel. The core stream was compressed horizontally by sheath flows on both sides (Figure 2a) and once it passed the chevrons, it was compressed vertically on the top and bottom. The maximum compression in vertical direction occurred right after the last chevron (Figure 2b). To improve the quality of the results, the quadratic solution was selected as the method of solving for the fluid dynamics part of the simulation. The boundary and initial conditions of the simulation is chosen to be similar to our experimental studies.

### 3.2. Experimental Results

Using this optofluidic cytometer, two different sizes of microspheres and three types of microalgae were characterized by investigating their forward light scatter. The signal output data set of the MPPC was saved using LabView software. The results demostrated that both width and height of the peaks for the larger microsphere were incresed. The number of data points collected for each peak was applicable to the sampling rate that was set on LabVIEW. The sampling rate of 100 K samples per second was chosen to maximize the number of data points collected for each microsphere. The power reading (2 μW) from the dump fiber was constantly monitored to make sure that all microspheres receive similar intensity of laser beam. A MATLAB code was developed to collect specific signal outputs produced by the samples. The code detects the peaks in the set of signals that are determined by an empirically defined threshold. The voltages greater than or equal to the threshold were saved for further analysis.

### 3.3. Characterization of Microbeads

**Figure 3:**
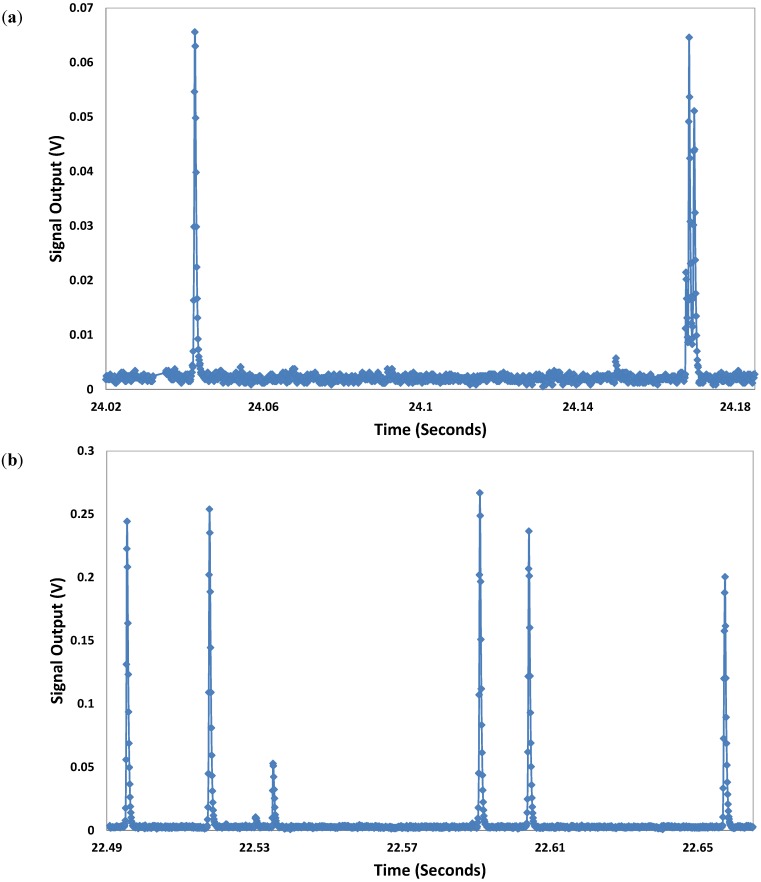
Voltage outputs produced by the (**a**) 3.2 μm and (**b**) 10.2 μm beads and collected by the MPPC. The signals collected for the 10.2 μm bead showed higher amplitude and width compared to those of the 3.2 μm bead. The 35 mW diode laser was used for excitation.

**Figure 4:**
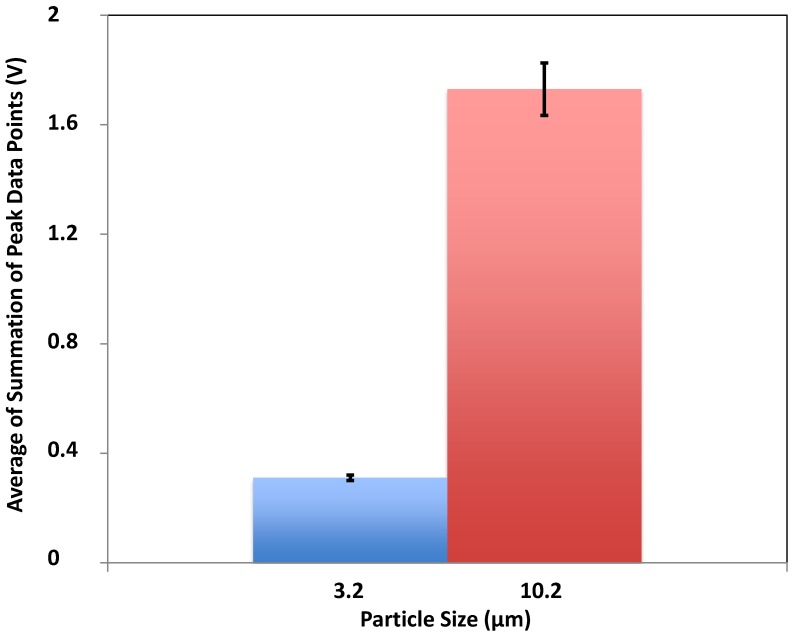
The statistical analysis for two different sizes of beads. The red bar shows higher average value for the summation of peak data points (SPDPs) for 10.2 μm particles compared to the average value calculated for 3.2 μm particles (blue bar). The average of SPDPs is 0.31 V for 3.2 μm and 1.71 V for 10.2 μm. The standard deviation is shown on each column.

We used a 635 nm diode laser (LAS-390-635-15, 35 mW, Lasermax Inc., Rochester, NY, USA) with 35 mW power output for characterization of the particles. The results revealed output signals with higher amplitude and width for the 10.2 μm beads compared to the 3.2 μm beads with similar experimental condition. These conditions include parameters such as sheath and sample volumetric flow rates, particle concentration, and type of photodetectors that were similar for both sets of experiments. Figure 3 shows the voltage outputs produced by the 3.2 and 10.2 μm beads and collected by the MPPC. The signals collected for the 10.2 μm bead showed higher amplitude and width compared to those of the 3.2 μm bead. Also, the number of data points collected for the 10.2 μm bead was larger bacause it takes slightly longer for the larger bead to pass through the interogation region where the light emission is collected by the multimode fiber. The dark noise rate (single photon equivalent) has produced about 0.004 V. Considering a linear relationship, the pulse produced by a 3.2 um bead should consist of about 15 photons. In the case of 10.2 um bead, there must be about 60 photons in each pulse. Figure 4 presents the statistical analysis for signal output collected for the beads. Each peak is generated by a bead passing through the interrogation region. The magnitutde of all data points collected for each peak is added and presented as a single value to represent each peak. This value is called summation of peak data points (SPDPs) in this paper. The blue and red bars show the average of these SPDPs. The averages for 10.2 and 3.2 μm beads were found to be 1.71 and 0.31 V, respectively.

The results show that for the bigger particles the average signal output is higher. The standard deviation for red and blue bars were 0.006 and 0.096, respectively. Integrating 3D hydrodynamiccal focusing design and a new generation of photodetector with high sensitivity and very small footprint, we were able to succesfully detect particles as small as 3.2 μm and discriminate populations of microparticles with different sizes.

### 3.4. Characterization of Microalgae

Three types of algae, *Chlamydomonas reinhardtii* strain 21 gr, *Chlamydomonas suppressor*, and *Chlorella sorokiniana*, were introduced into the microchannel so the signal output from each sample could be assessed. Since the microalgae have different shapes and sizes, signals with different amplitude and width were collected for each type of cell. *Chlamydomonas reinhardtii* strain 21 gr is the most popular type of green algae and it is widely used in laboratory research. It is also called *Chlorophyta* and is oval in shape. This strain can be found in many sources such as fresh water, soil, and oceans. It has a cell wall, an eye that senses the light, a chloroplast and two flagella. *Chlamydomonas reinhardtii* strain 21 gr and *Chlamydomonas suppressor* are different in size, but have similar physical features. *Chlorella sorokiniana* is a type of green microalgae that can be found in fresh water and is being used to produce biodiesel fuels. It is spherical in shape and 2–10 microns in diameter. It has no flagella and reproduces at a very fast rate.

**Figure 5:**
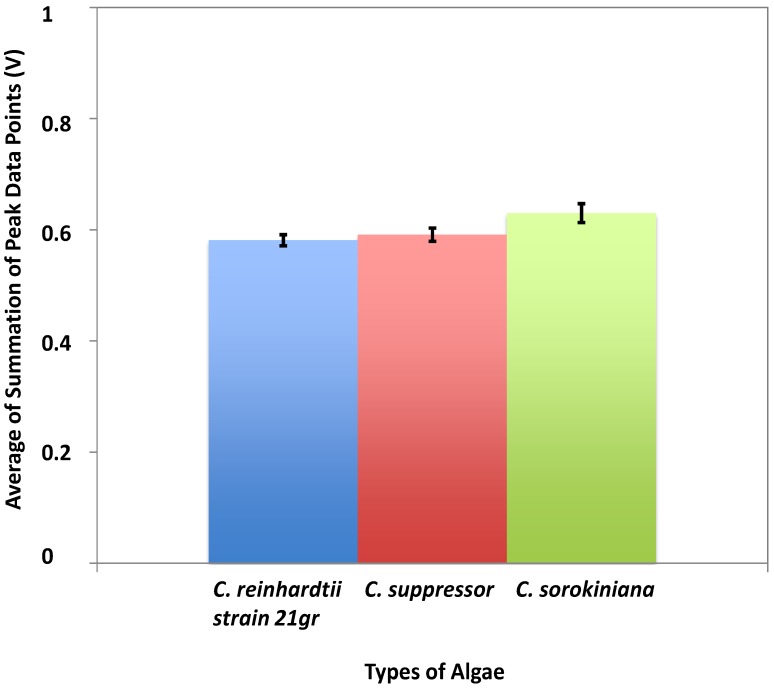
The statistical analysis for three types of algae. The blue bar shows the average of SPDPs for *Chlamydomonas reinhardtii* strain 21 gr, red bar represents the average value for *Chlamydomonas suppressor*, and green bar shows the average value for *Chlorella sorokiniana*. The average of SPDPs is 0.58 V for *Chlamydomonas reinhardtii* strain 21 gr, 0.59 V *Chlamydomonas suppressor*, and 0.63 V for *Chlorella sorokiniana*. The standard deviation is shown on each column.

The signal output collected for *Chlamydomonas reinhardtii* strain 21 gr is very similar to *Chlamydomonas suppressor* while the signal output of *Chlorella sorokiniana* clearly has a higher magnitude compared to other two types of algae. This confirmed that *Chlorella sorokiniana* are larger compared to the other two types. Microscopy images confirmed these results with respect to the sizes of the algae. We were able to successfully detect the slight difference in size for *Chlamydomonas reinhardtii* strain 21 gr and *Chlamydomonas suppressor* using further statistical analysis as shown in Figure 5.

For *Chlorella sorokiniana*, the signal output is clearly higher than that of the *Chlamydomonas suppressor* and *Chlamydomonas reinhardtii* strain 21 gr. This proves that *Chlorella sorokiniana* is larger in size compared to other two types of algae. *Chlamydomonas reinhardtii* strain 21 gr was found to be slightly smaller than *Chlamydomonas suppressor*, which was confirmed by the microscopy images. The average of SPDPs for *Chlorella sorokiniana* is 0.63 V, which is 0.04–0.05 V (about 8%) larger than the average values produced by other two types of algae. Comparing statistical analysis for algae and beads revealed that the averages of SPDPs for algal samples fall between the 0.3 and 1.71 V that is found for the 3.2 and 10.2 μm beads. The microscopy images of the algae also show that majority of microorganisms are smaller than 10 μm.

## 4. Conclusions

We have designed and developed an optofluidic flow cytometer equipped with a MPPC. The high sensitivity of the MPPC photodetector allowed us to detect particles in the range few microns precisely. Our new optofluidic system has a significantly smaller footprint as opposed to the previous generation of flow cytometers with the photomultiplier tubes as their main photo detection units. Using our system, we analyzed samples of microalgae such as *Chlamydomonas reinhardtii* strain 21 gr, *Chlamydomonas suppressor*, and *Chlorella sorokiniana* as well as microspheres. This device proved sensitive enough to detect particles in the range of 3–10 μm. The results showed that larger particles/cells produced signals with larger widths and heights while passing through the interrogation region. Our COMSOL simulation results confirmed the 3D hydrodynamic focusing of the core stream by the sheath stream and arrays of chevrons. The next step for the design of the microfluidic system could be to incorporate an imaging system into the optical train. With the integration of an imagining device the samples could be analyzed more precisely, which would be beneficial in point of care diagnostics and for research purposes. Another beneficial improvement to the flow cytometer design would be reducing the power requirement. This would be specifically important for point of care analysis.
